# Supplementary material for: Self-care interventions to assist family physicians with mental health care of older patients during the COVID-19 pandemic: Feasibility, acceptability, and outcomes of a pilot randomized controlled trial
Source: PLoS One. 2024 Feb 15;19(2):e0297937. doi: 10.1371/journal.pone.0297937 (PMC10868770; doi:10.1371/journal.pone.0297937)
Supplement: S1 Appendix — (PDF) [file pone.0297937.s002.pdf]

Appendix 1

Description of the PanDirect self-care tools

Yaffe et al., Self-care interventions to assist family physicians with mental health care of older patients during the COVID-19 pandemic: Feasibility, acceptability, and outcomes of a pilot randomized controlled trial

Based on the specific mental health symptoms identified on the PHQ-9 and GAD-7 at baseline, the study coordinator applied an algorithm developed for this study that assigned specific self-care tools to each participant. Each participant was assigned one primary tool, and one secondary tool. Each tool was accompanied with a cover sheet which provided an overview and very basic instruction. Cover sheets are provided in this appendix, **references for the tools appear at the bottom of the cover sheets and in the manuscript**. French versions of the tools were available for French-speaking participants and are available upon request from the corresponding author.

Primary tools (i.e. the workbook). Participants received either:

- The Reactivating Your Life chapter from the Antidepressant Skills Workbook (p.2)
- The Managing Worry chapter from the Positive Coping With Health Conditions Workbook (p.3)

Secondary tools. Participants received one of the following options, based on identified needs:

- The mood monitoring tool (p.4)
- A relaxation audio tool (p.5)
- Information on exercise and healthy eating (p.6)
- Information on sleep (p.7)
- Information on emotional eating (p.8)

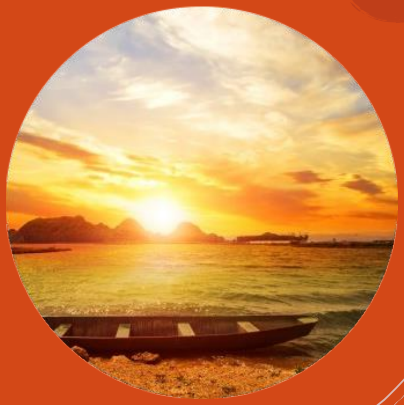

REACTIVATING YOUR LIFE WORKBOOK

When we are feeling down, worried, lonely and/or tired, we are less likely to engage in activities and hobbies that we normally enjoy. Unfortunately, the less we do these activities, the worse we can feel.

The enclosed workbook contains 5 sections to help you gradually increase pleasurable activities in your life by:

1. Identifying activities to increase
2. Choosing two of these activities
3. Setting realistic goals
4. Carrying out your goals
5. Reviewing your goals

Please note that while these sections mention depression, we believe they can be useful for anyone experiencing stress, tension, low mood, feelings of loneliness and/or fatigue

We suggest reading through these sections over the next few weeks and trying the exercises gradually as you go through the workbook. This may allow you to gradually increase your daily activity level, mood, motivation, energy and overall wellbeing.

Information reprinted with permission:
Bilsker, D., & Paterson, R. (2005).
The antidepressant skills workbook: Self-care depression program.
2nd edition. Vancouver, BC: BC Ministry of Health

panDIRECT

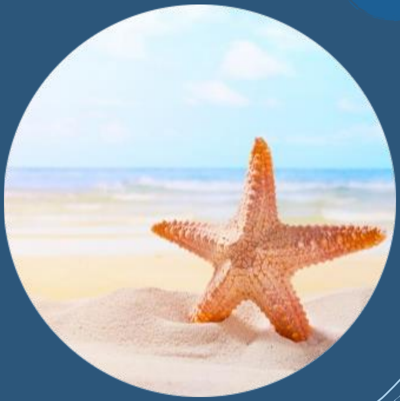

MANAGING WORRY WORKBOOK

Worrisome thoughts and anxious feelings can be challenging to cope with. They can also bring about other difficulties, such as trouble sleeping, over- or undereating, restlessness and/or tension. This workbook is intended to help you manage unpleasant or worrisome thoughts and practice having calmer thoughts.

The workbook contains 5 sections to address the thoughts you may be experiencing by:

1. Identifying worry thoughts
2. Challenging worry thoughts
3. Practicing calming and realistic thinking
4. Scheduling worry time
5. Using methods to protect your sleep

We suggest reading through the material over the next few weeks and practicing the suggested exercises if you find them appropriate for you. This may allow you to better target your worrisome thoughts and reduce feelings of nervousness, restlessness or anxiety.

Workbook reprinted with permission:
Bilsker, D., Samra, J., & Goldner, E. M. (2009).
Positive coping with health conditions: A self-care workbook.
Consortium for Organizational Mental Healthcare (COMH).

pandirect

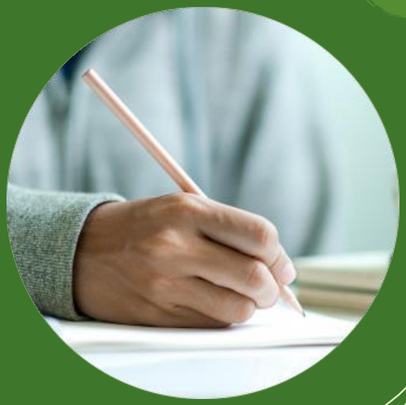

MOOD MONITORING JOURNAL

Everyone experiences changes in their moods. Sometimes moods can be relatively stable over a longer period of time and other times moods can shift quickly throughout the day. It can be useful to track our moods to understand how our feelings change and what triggers those changes. We can then use this information to manage our moods and feel better faster.

We recommend trying the enclosed mood monitoring journal for at least a week to see if it is helpful for you. It only takes a few minutes each day to complete. Some people find it useful to keep the journal next to their bed or on their refrigerator to remember to complete it. Others prefer to set reminders or keep their notes on their telephone. Try different ways to incorporate it into your daily routine.

If you find it beneficial, we encourage you to keep using it regularly and see if you can continue to draw links between your moods and the situations or events in your daily life.

Mood monitoring journal prepared in accordance with recommendations in:
Wagner EH, Austin BT and Von Korff M (1996)
Organizing care for patients with chronic illness.
Milbank Quarterly 74(4): 511–544;
and based on principles in:
Beck AT. *Cognitive Therapy of Depression*.
The Guilford Press: New York, 1979

panDIRECT

RELAXATION AUDIO RECORDINGS

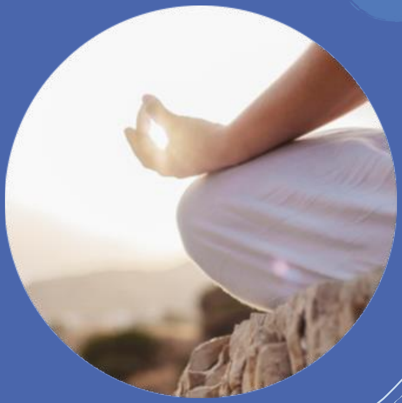

Guided relaxation can be particularly helpful if you are experiencing stress, anxiety, or difficulty sleeping. We have included several audio recordings to guide you through various relation exercises.

You can find some of these exercises on this website: https://trilliumhealthpartners.ca/stayinghealthy/Pages/Relaxation-Training.aspx

On this site, you will see the following exercises:

1. General relaxation instructions
2. Relaxed breathing
3. Progressive muscle relaxation
4. Guided imagery

We encourage you to listen to and practice all of these exercises since they complement each other, and together promote greater relaxation. We suggest listening to the general relaxation instructions first, especially if you have not tried guided relaxation in the past. Relaxed breathing can be a useful tool to reduce stress, and it can easily be practiced on a regular basis if you find it helpful. Progressive muscle relaxation and guided imagery can be used to increase feelings of relaxation and decrease physical tension. They can equally be great to help you sleep if you’re having difficulties falling or staying asleep at night.

In addition, we have included a CD with recordings that can help you decrease anxiety and increase overall wellbeing.

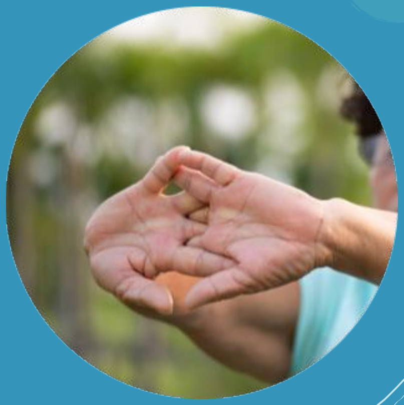

DIET & PHYSICAL ACTIVITY

Research suggests that adopting a healthy, active lifestyle has many health benefits. Eating well and engaging in light to moderate exercise can boost your mood, improve your sleep and decrease stress and fatigue.

We have included two information sheets with strategies to incorporate healthy eating and physical activity in your daily routine:

1. Diet information sheet
2. Physical activity information sheet

Please note that while these sheets mention depression, we believe they can be useful for anyone experiencing tension, low mood, worry, reduced energy levels and/or sleep difficulties.

In addition to reading through these two sheets over the next few weeks, we recommend looking at Canada's Food Guide for an idea of the portions and types of food to consume on a daily basis:

https://food-guide.canada.ca/en/guidelines/appendix-b-summary-of-guidelines-and-considerations

Information reprinted with permission:
Bilsker, D., & Paterson, R. (2005).
The antidepressant skills workbook: Self-care depression program.
2nd edition. Vancouver, BC: BC Ministry of Health.

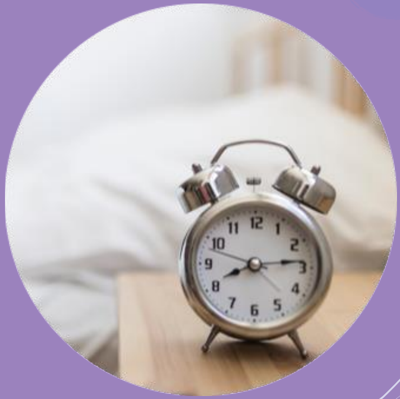

SLEEP DIFFICULTIES

It can be frustrating to experience trouble falling asleep and/or staying asleep. Sleep difficulties often lead to increased daytime tiredness, which can in turn have an effect on mood, on interests in activities and hobbies, and on daily functioning at work or at home.

The enclosed information sheets contain tips to improve your quality of sleep and draw awareness to your use of coffee, tea, and other caffeinated products, as this can be one of the main causes for sleep issues:

1. Sleep information sheet
2. Caffeine information sheet

Please note that while these sheets mention depression, we believe they can be useful for anyone experiencing anxiety, stress or tension, low mood, reduced energy levels and/or sleep difficulties.

We suggest reading through these sheets over the next few weeks and trying to implement some of the suggestions in your daily routine if you find them appropriate for you. It may take several days or weeks to notice a difference in your energy level, and it's normal to feel more tired when you begin to decrease your caffeine intake or make changes to your sleeping habits. Over time, you should see improvements in your energy level and overall feelings of wellbeing.

Information reprinted with permission:
Bilsker, D., & Paterson, R. (2005).
The antidepressant skills workbook: Self-care depression program.
2nd edition. Vancouver, BC: BC Ministry of Health.

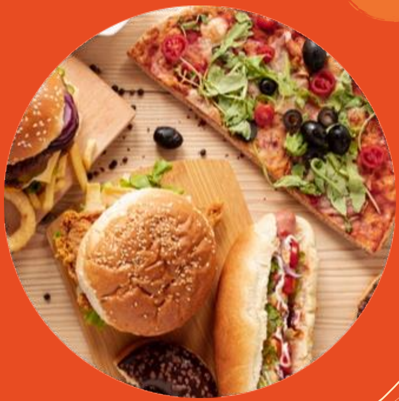

EMOTIONAL EATING WORKSHEET

Stress, mood fluctuations, anxiety, fatigue, and boredom can lead us to eat more or reach for unhealthy food. While eating may temporarily soothe us or distract us from our problems, it can trigger feelings of guilt and shame afterwards. Regularly eating too much or consuming unhealthy food can also lead to physical problems long-term.

The Emotional Eating worksheet contains five sections that can help you to better understand, identify, and manage emotional eating by:

1. Understanding the connection between food and mood
2. Identifying eating triggers
3. Differentiating between emotional and physical hunger
4. Managing emotional eating
5. Coping with difficult emotions

We suggest reading through these sections during the next few weeks and trying the suggested techniques as you go through the worksheet. We also recommend consulting Canada's Food Guide for an idea of the portions and types of food to consume on a daily basis:

https://food-guide.canada.ca/en/guidelines/appendix-b-summary-of-guidelines-and-considerations

Information adapted from the
Hamilton Family Health Team Registered Dietitians
and from www.AmIHungry.com
