# Supplementary material for: Self-care interventions to assist family physicians with mental health care of older patients during the COVID-19 pandemic: Feasibility, acceptability, and outcomes of a pilot randomized controlled trial
Source: PLoS One. 2024 Feb 15;19(2):e0297937. doi: 10.1371/journal.pone.0297937 (PMC10868770; doi:10.1371/journal.pone.0297937)
Supplement: S2 Appendix — (PDF) [file pone.0297937.s003.pdf]

Appendix 2: Detail of multiple imputation approach and additional analyses on patient outcomes

The analysis by intention-to-treat was performed by following these two steps. The first step was to investigate the mechanism of missing values to identify meaningful baseline characteristics. This was done by computing for each baseline characteristics the standardized difference (SD) between 2-month survey completers and non-completers; any baseline variables with a SD ≥0.15 were considered clinically relevant (see Table A). The second step was to conduct Multiple Imputation (MI) approach using meaningful covariates to loss of follow-up. Because of the small sample size (n=90), up to 9 clinically relevant variables (see Table A) were selected for MI to respect a ratio of 10 observations per variable (Harrell, 2001). The variables with the highest value of SD were first selected; for every variable with more than 2 categories, each additional category needs to be counted as an additional variable. For each outcome, MI approach was used under the Missing at Random (MAR) assumption to impute 2-month missing outcome; imputation by chained equations (MICE) was performed. The MICE uses a separate conditional distribution for each imputed variable and no assumption of multivariate normality is required. (Rubin, 1987, Buuren, 2010).

Additional analyses on completers of the 2-month survey for the two patient outcomes were presented in Table B. Five additional regressions models were fitted, adjusted for the same set of variables in Table 4 and including one by one the variable showing imbalance between the two study groups from the list of variables not selected a priori (see Table 1). Same methodology presented in the data analysis section was used to compute effect sizes and 95% confidence intervals.

References

Buuren, S. 2010. Multiple imputation of discrete and continuous data by fully conditional specification. *Statistical Methods in Medical Research***,** 16.

Harrell, F. E. J. 2001. *Regression Modeling Strategies With Applications to Linear Models, Logistic Regression, and Survival Analysis,* New York, NY, Springer.

Rubin, D. B. 1987. *Multiple imputation for nonresponse in surveys,* New York, John Wiley & Sons.

Table A: Patient characteristics by 2-month completers vs non-completers (n=90)					
	Completers (n=62)		Non-completers (n=28)		Standardized
Variables	n	%	n	%	difference
**Categorical variables:**					
Study group					0.24
Intervention	28	45.2	16	57.1	
Control	34	54.8	12	42.9	
PHQ-9					0.47
0-4	32	51.6	20	71.4	
5-9	15	24.2	4	14.3	
10-14	7	11.3	3	10.7	
15+	8	12.9	1	3.6	
GAD-7					0.25
0-4	43	69.4	22	78.6	
5-9	14	22.6	4	14.3	
10+	5	8.1	2	7.1	
Age					0.66
65-74	21	33.9	3	10.7	
75-84	30	48.4	14	50.0	
85+	11	17.7	11	39.3	
Sex					0.01
Female	35	56.5	16	57.1	
Male	27	43.5	12	42.9	
Education					0.93
University degree	34	54.8	6	21.4	
High school and post	15	24.2	5	17.9	
Completed high school	8	12.9	10	35.7	
Less than high school	5	8.1	7	25.0	
Presence of a caregiver					0.17
No	22	35.5	12	42.9	
Yes-does not live with	23	37.1	9	32.1	
Yes-live with	17	27.4	7	25.0	
CAGE score					0.24
0	54	90.0	24	96.0	
1+	6	10.0	1	4.0	
(missing)	(2)		(3)		
Receiving homecare services from the CLSC					0.45
No	51	86.4	19	67.9	
Yes	8	13.6	9	32.1	
(missing)	(3)		(0)		
Previous counseling since March 2020					0.10
No	55	94.8	24	92.3	
Yes	3	5.2	2	7.7	
(missing)	(4)		(2)		
ED visits without hospitalization*					0.01
No	55	88.7	23	88.5	
Yes	7	11.3	3	11.5	
(missing)	(0)		(2)		
Hospitalization*					0.31
No	49	79.0	17	65.4	
Yes	13	21.0	9	34.6	
(missing)	(0)		(2)		

Table B: Additional results of 2-month outcomes;
effect size and 95% confidence interval of study group (n=62)

	2-month outcomes:					
**Informations**	**PHQ-9**			**GAD-7**		
**Completers:**	**Effect size**	**95% CI**	**p-value**	**Effect size**	**95% CI**	**p-value**
Model adjusted for:						
1- Baseline imbalance and education	0.08	[-0.30; 0.46]	0.677	0.02	[-0.33; 0.35]	0.934
2- Baseline imbalance and presence of caregiver	0.08	[-0.29; 0.46]	0.651	-0.09	[-0.41; 0.26]	0.650
3- Baseline imbalance and CAGE score	0.18	[-0.19; 0.55]	0.340	0.00	[-0.33; 0.34]	0.992
4- Baseline imbalance and counseling/help	0.18	[-0.18; 0.55]	0.315	-0.02	[-0.35; 0.31]	0.900
5- Baseline imbalance and hospitalisation	0.12	[-0.25; 0.48]	0.512	0.00	[-0.33; 0.33]	0.990

Baseline imbalance: PHQ-9, GAD-7, age group, sex
