# Supplementary material for: Self-care interventions to assist family physicians with mental health care of older patients during the COVID-19 pandemic: Feasibility, acceptability, and outcomes of a pilot randomized controlled trial
Source: PLoS One. 2024 Feb 15;19(2):e0297937. doi: 10.1371/journal.pone.0297937 (PMC10868770; doi:10.1371/journal.pone.0297937)
Supplement: S1 Protocol — (PDF) [file pone.0297937.s004.pdf]

ASSISTING FAMILY PHYSICIANS WITH GAPS IN MENTAL HEALTH CARE GENERATED BY THE COVID-19 PANDEMIC

Principal Investigator:

Mark Yaffe, MD
Professor, Department of Family Medicine, McGill University
Family physician, St. Mary's Hospital Center
3830 Lacombe Ave.
Montreal (QC) H3T 1M5
Tel: 514-734-2676
e-mail: mark.yaffe@mcgill.ca

Co-Investigators:

Jane McCusker, MD DrPH
Professor Emerita, Department of Epidemiology, Biostatistics and Occupational Health, McGill University
Principal Scientist, St. Mary's Research Centre

Sylvie Lambert, PhD
Associate professor, Ingram School of Nursing, McGill University
Research Associate, St. Mary's Research Centre

Jeannie Haggerty, PhD
Professor, Department of Family Medicine, McGill University
McGill Research Chair in Family & Community Medicine and Principal Scientist, St. Mary's Research Centre

Ari N. Meguerditchian, MD
Scientific Director, St-Mary's Research Center
Associate Professor Surgery & Oncology, McGill University
Surgical Oncologist, MUHC

Marc Pineault, MSc
Coordinator – access to primary care services and specialized medicine, Professional Services Directorate, CIUSSS Montreal West Island

Funding Agency:

Foundation for Advancing Family Medicine of the College of Family Physicians of Canada and Foundation of Canadian Medical Association
COVID-19 Pandemic Response & Impact Grant Program (Co-RIG)

Date: December 16, 2021

Table of contents

1. BACKGROUND 3
1.1 Pandemic rapid response. 3
1.2 Mental health of older adults and their caregivers during the COVID-19 pandemic. 3
1.3 Telephone supported self-care interventions for mental health problems. 3
2. RESEARCH OBJECTIVES 3
3. METHODS 4
3.1 Study Design and timeline 4
3.2 Sample and exclusion criteria 4
3.3 Recontact and consent 5
3.4 Data collection from patient participants 5
3.5 Interventions 6
3.6 Randomization 7
3.7 Follow-up of at risk participants 7
3.8 Communication with FPs 8
3.9 Data analysis and statistical power 8
4. CONFIDENTIALITY OF PARTICIPANT DATA 9
5. REFERENCES 9
6. APPENDICES 13
Appendix 0: Information on the 2-1-1 hotline 13
Appendix A: Recruitment script and screening questions 13
Appendix B: Consent form 13
Appendix C: Baseline and follow-up questionnaire 13
Appendix D: Toolkit contents, description of the algorithm 13
Appendix E: Coach manual 13
Appendix F: Fidelity checklist 14
Appendix G: Summary report for family physicians 14
Appendix H: Survey for family physician 14

1. BACKGROUND

1.1 Pandemic rapid response.

The Foundation for the Advancement of Family Medicine and the Foundation of the Canadian Medical Association have identified an urgent need to develop novel approaches to addressing outcomes of the COVID-19 pandemic. They are consequently financing the Co-RIG Program Phase 1, designed to support practice innovations that reduce the direct or indirect harms of COVID-19 and target family practice innovative interventions that are likely to have a measurable, positive impact on patients and/or populations within 6 months of the start of funding. This protocol is a response to that mandate.

1.2 Mental health of older adults and their caregivers during the COVID-19 pandemic.

During the COVID-19 pandemic, older adults with chronic physical conditions are a particularly vulnerable population for unmet mental health needs.[1-5] Depression and anxiety are several-fold more prevalent in this population in comparison to counterparts without chronic conditions, and may be even higher during the pandemic.[6-10] The pandemic containment measures are also likely to exacerbate pre-existing problems with detection of mental health conditions in older adults.[8, 11, 12] Anxiety appears to be the most prevalent emotional response within the pandemic.[8, 12, 13]

1.3 Telephone supported self-care interventions for mental health problems.

With the COVID-19 pandemic, there has been a rapid deployment of telehealth. For older adults, the telephone remains the most reliable telehealth modality,[14] because they experience more difficulty and make more errors in completing online tasks than their younger counterparts due to age-related cognitive and motor function impairments.[15, 16] Telephone-supported strategies are a promising avenue for expanding access to mental health care for older adults with chronic physical conditions, with potential for increasing quality of life, reducing healthcare expenditures, and promoting independence in activities of daily living.[17]

Worldwide, self-care interventions are recommended as the first level in a stepped care program for managing and treating mild-to-moderate depression and anxiety.[18, 19] Meta-analyses affirm that guided self-care interventions and face-to-face psychological therapies for depression and anxiety have comparable effects,[20] and that supported (or guided) self-care is more effective than unsupported.[21] Our team has conducted two RCTs of telephone-supported mental health self-care interventions comprising a cognitive-behavioral therapy (CBT) skills-building toolkit and lay telephone coaching. The RCTs concluded that, compared to the self-care toolkit alone, telephone guidance from a lay coach improved adherence[22] and clinical outcomes in middle-aged and older adults with chronic physical conditions and comorbid depressive symptoms (DIRECTsc).[23] Among cancer survivors with depression frequently accompanied by anxiety (CanDIRECT), a similar guided self-care intervention improved 6 month depression and anxiety symptoms, as well as mental health-related quality of life.[24,25] Given this strong evidence base and the success of deploying the intervention in two large, funded RCTs, our proposal is to evaluate the delivery of a shortened, adapted intervention in real-world conditions in a cohort of vulnerable community-based older adults (65+ years).

2. RESEARCH OBJECTIVES

We will assess and compare in a Randomized Control Trial (RCT) two self-care intervention strategies with 8-week follow-up:

1. The feasibility of delivering the interventions during the pandemic;

2. The acceptability of the interventions for patients;
3. The acceptability of the interventions for family physicians.
4. The effectiveness of the interventions on severity of symptoms of depression and anxiety at 8 weeks.

Criteria for success for feasibility include measures of recruitment, retention and fidelity of intervention delivery: at least 75% of patients will agree to participate; at least 75% will complete the 2-month follow-up and at least 85% of the intervention components will be delivered as per the intervention protocol (see description of checklist further below). Success regarding acceptability outcomes: at least 75% will use the self-care tools; at least 75% of those assigned to receive coaching will complete 3 calls; and at least 75% in both groups will have a positive satisfaction score at 2 months (Client Satisfaction Questionnaire (CSQ) score of at least 75%).

3. METHODS

3.1 Study Design and timeline

We will conduct a single blind, individually randomized, pilot trial of 2 self-care strategies to address symptoms of depression and anxiety (either premorbid or resultant from the pandemic) among home-based seniors with chronic physical conditions. The strategies to be tested are 1) validated self-care tools (internet and paper-based) plus up to 3 telephone coach calls; 2) self-guided tools alone. The trial design adheres to the CONSORT criteria,[26] the CONSORT extension for pragmatic trials,[27] and guidelines for behavioral trials.[28] The trial protocol is registered on clinicaltrials.gov.

Recruitment and follow-up will be conducted until the end of 2020, with results to be reported in early 2021. As mentioned in section 1.1, the funders have targeted immediate and short-term innovations that maximize the effectiveness of care for patients and are looking for projects to be completed within a 6-month timeframe.

3.2 Sample and exclusion criteria

We have already assembled a cohort of 267 patients 65+ years old for a previous study (PCAP study, see SMRC protocol #18-04).[29] Among these, 80% (n=213) agreed to be contacted again for future research opportunities. These patients were discharged home following hospitalization between October 2018 and December 2019 at the medical or geriatric units of two general hospitals on the west island of Montreal. Among the original cohort, 40% were born outside Canada; 30% received homecare services, and 65% were high-risk for functional decline, death, and increased health care service utilization.[30]

With anticipated 75% participation, we project a sample size of 160. Exclusion criteria for the current study will comprise: moderate to severe cognitive impairment (using BOMC a brief cognitive screen) [31, 32]; unable to read in English or French (self-reported); hearing impairment (as judged by research staff); currently receiving counseling or psychological therapy (as these treatments may conflict with the self-care interventions) [33]; currently living in a long term care or other medicalized facility, or presenting suicidal intent (as identified through the final item of the PHQ-9 which will be asked at screening). Patients with suicidal intent will be contacted by the coach supervisor for further assessment and referral (see section 3.7 below). Patients receiving antidepressant or anxiolytic medications will be eligible for the study, as will all patients who do not meet the threshold for clinically-significant symptoms of depression and/or anxiety as they

may still benefit from the intervention Participants who begin counseling or psychological therapy after enrolment will not be withdrawn. Participants who are not eligible or interested in the study will be invited to contact 2-1-1 to follow-up on any needs (see www.211qc.ca/en/).

3.3 Recontact and consent

Research assistants (RAs) will contact this cohort by telephone to screen for eligibility and invite them to participate in the proposed study (see script and screening questions in Appendix A). Interested, eligible patients will be invited to provide consent over the telephone (see consent form in Appendix B). The RA will read the consent form to the patient, answering questions and providing clarification as needed. The RA will document consent on the form and will sign the form. A copy of this signed consent form will be mailed to participants or sent electronically following the consent process. The telephone call will be audio-recorded for quality control and to ensure that a, audio record of the verbal consent process is saved. If participants prefer to view the consent form prior to screening, a copy will be sent by mail or electronically and a follow-up call will be made to re-assess interest.

We are seeking to obtain verbal consent because written consent may not be feasible in the current context, with postal service delays. Participants may not be able to easily return signed consent forms through the mail. As mentioned above, the project must be completed within a 6-month timeframe, with funders recognizing that there is an urgent need among the target population and a need to assist family physicians to handle anxiety and depression symptoms during and after pandemic waves. Having to wait for consent forms to be returned before proceeding may lead to significant delays. The study and intervention are very low risk, with the self-care tools having already been tested in full RCTs and with participants, having already completed the study described in section 3.2, already being familiar with standard study procedures. Participants will have a mailed copy for their records to refer back to as needed.

3.4 Data collection from patient participants

Following consent, RAs will conduct the baseline interview (see Appendix C) with participants. Measures to be collected are as follows:

- Symptoms of depression: We will use the PHQ-9, a widely used 9-item measure of depression severity to determine presence and type of depressive symptoms.[34] The PHQ-9 was selected based on its wide use in medical populations,[35] established severity ranges and sensitivity to change,[36] validation as a screening measure,[37] and inclusion of somatic symptoms which are recognized as a core domain of depression, not confounding diagnosis in patients with chronic disease.[38, 39] The criterion for clinically-significant depression is 10 (but as specified above, this is not being used as an eligibility cut-point).
- Severity of anxiety symptoms: The seven item GAD-7 will be used as a continuous measure of severity of anxiety symptoms (internal consistency: Cronbach $\alpha = .92$; test-retest reliability: intraclass correlation=0.83).[40] Scores range from 0-21. The criterion for clinically-significant anxiety is 10 (also not used as an eligibility cut-point)
- Alcohol abuse: The 4 item CAGE questionnaire with a score ranging from 0-4 will be used to detect alcohol abuse and dependence. Although a cut point of 2 or more is used conventionally (average sensitivity 0.71, specificity 0.90), the cut point of 1 or more results in greater sensitivity as expected, and has been used in some studies.[41] We will categorize as: no, possible (score of 1), or probable (score of 2+).

- Other baseline data: include questions on hospitalizations and ED visits during the previous 6 months, COVID diagnoses in patient or family, current living arrangement, having a family caregiver, and current homecare services.

At 8 weeks, with an anticipated follow-up rate of 75% (120 end of project interviews), the PHQ-9, and GAD-7 will be re-administered, along with enquiry into use of the self-care materials, perceived helpfulness in managing mental health problems, satisfaction with the intervention, and use of health services including any counseling or therapy started, during the 8-week study period. Satisfaction will be measured with a 3-item version of the Client Satisfaction Questionnaire (CSQ-3),[42, 43] highly correlated with the CSQ-8 (r=0.94).

Additionally, RAs, coaches and the coach supervisor will carefully track information required to address the feasibility objective using dedicated follow-up logs and checklists.

3.5 Interventions

The interventions to be evaluated in the RCT are: 1) the algorithm-determined self-care tools, matched to the specific mental health symptoms reported by participants, and 2) the tools plus up to 3 coach calls. All participants receive the self-care tools; only one group receives the coaching. This design allows us to evaluate the feasibility and acceptability of the tools, and the additional benefit (if any) of the coaching. Coaching is expected to increase use of the tools.

The tools and the coaching manual will be adapted from those we have successfully deployed in the DIRECTsc depression self-care project focusing on patients with depressive symptoms,[23] but abbreviated to meet the needs of the proposed short-term intervention for a broader sample of patients to include those with anxiety symptoms and with minimal symptoms. Tools will include individual chapters of the Antidepressant Skills Workbook;[44, 45] the mood monitoring tool;[23, 24] a workbook on managing worry;[46] relaxation audio files and information on exercise and healthy eating (see Toolkit contents described in Appendix D). In view of the short duration of the intervention (8 weeks), a maximum of 2 tools will be sent to each participant.

An algorithm will determine which self-care tools, matched to the specific mental health symptoms reported by participants, will be couriered to the participants. The study coordinator will apply the algorithm (using a paper form) to the participant's baseline results. An administrative assistant will be available at the Research Centre to assemble the required tools and arrange for them to be couriered to participants' homes using a secure courier service we have used in previous studies. Along with the tools, participants will be sent a welcome letter describing their study group assignment, and what to expect next (coach calls, if relevant, and expected timeline for the follow-up questionnaire).

Coaching by a trained lay coach will be structured and guided by a manual. Trained lay coaches will call participants in the week following delivery of the toolkit to guide them through the self-care toolkit over an 8-week period. Coaches will contact participants a maximum of 3 times, with calls expected to average 15-20 minutes. Call content will be guided by a structured coaching manual adapted from those used in our previous two RCTs.[23, 47] The coaches will follow structured agendas, keep records of all contacts, and will be trained to follow-up on any reported suicidal thoughts and to identify if there is intent to act, using a previously-developed protocol (which includes referring these participants to the coach supervisor for proper follow-up, see section 3.7 below).[23, 25]

The coaches will be bilingual (English, French), with excellent organizational and interpersonal skills, but without formal training in CBT (e.g., psychology undergraduate students). In previous

research, we have successfully used both psychology undergraduate students and others. The personal characteristics and maturity of the individual will be the most important criteria used to select coaches. They will be trained during a half-a day training session led via Zoom, based on the Coach Manual (see Appendix E - Coach manual) and mock practice sessions conducted over the telephone. Coaches will be trained and supervised by the coach supervisor, Alexandra Barnabé (psychotherapist and PhD candidate, McGill Clinical Psychology, fully bilingual). AB is familiar with the DIRECT-sc intervention, has worked with co-investigators previously, and has been involved with planning the adaptation of the intervention to suit the current study. Her previous experience with intervention development and student supervision are matched to the requirements for the coach supervisor, and her experience working as a Psychosocial Intake Counselor and Crisis Management Services Coordinator will allow her to follow-up with participants who may express suicidal ideation. She is registered in the student and doctoral candidates' registry of the Ordre des Psychologues du Quebec, which permits her to directly assess and provide intervention to patients. Alexandra's clinical PhD supervisor is available should she need to discuss participant follow-up or referral in the context of this current study.

The coach supervisor will attend monthly investigator meetings. She will use a fidelity checklist (adapted from one used in our previous research, see Appendix F - fidelity checklist) and will also blindly enter coaching data into blank copy of the coach log to monitor all coach contacts initially, until a satisfactory level of adherence to the directives in the coaching manual is attained. After this, s/he will monitor a sample (selected by the statistician) of calls using the checklist. Any discrepancies between the log of the coach and the log of the coach supervisor will be reviewed with the coach supervisor and the coach.

The coaches will record in their logs all contacts attempted and completed, duration of contacts, and tools that were recommended and/or used. Additionally, the coach will record whether the participant is setting goals independently or with prompting from the coach.

3.6 Randomization

Participants will be randomized to Group 1 (tools only) or Group 2 (tools with coaching) by a computer-generated randomization schedule that uses random block sizes, with an allocation ratio of 1:1; SAS University Edition will be used. In order to ensure allocation concealment, the study coordinator will first verify eligibility and informed consent. Subsequently, the unique patient identifier (PID) from the recruitment log will be entered into the randomization schedule, along with: date of consent, date of baseline interview. An automated interface will then assign the participant to the study group. Participants will be stratified based on whether their symptoms are none to mild or moderate to severe to ensure an equal balance of symptom severity in both study groups. Participants will be categorized as none to mild if their PHQ-9 is less than 10 and their GAD-7 score is less than10. Participants with a PHQ-9 score of 10 or more and/or a GAD-7 score of 10 or more will be categorized as being in the moderate to severe group.

3.7 Follow-up of at risk participants

All research staff members who have patient contact are trained to follow-up on any reported suicidal thoughts to identify if there is intent to act. All patients who report only thoughts, but no intent, will be given the telephone numbers of local crisis/suicide hotlines. The coach supervisor will follow-up immediately with any participant who divulges suicidal intent. If the patient is deemed at risk, the patient's most responsible physician (as identified by the participant at that time) will be informed with the patient's consent. Furthermore, if the coach supervisor identifies any immediate risk of self-harm, the patient will be directed to the nearest emergency room, will

be given the telephone numbers of a suicide hotline and the most responsible physician will be informed (the police will be alerted if the patient with suicidal behaviours refuses to go to the emergency room). Those with suicidal intent identified at screening will be excluded from the study; those with suicidal intent during follow-up will be withdrawn from the study.

Participants with a PHQ-9 score of 20 or more will be encouraged to see their physician. If patients consent, the identified physician will be informed.

3.8 Communication with FPs

We are partnering with the Integrated University Centre for Health and Social Services of West Island of Montreal (IUCHSS). It will be represented by Co-I Marc Pineault. We have partnered with 4 Family Medicine Groups (FMGs) in our previous projects and will reach out to them again (in collaboration with Marc Pineault) as we prepare to implement this new project.

If patients consent (see Appendix B), family physicians will receive a summary report of their patients' involvement in the study (see Appendix G). The report will include information regarding the presence of specific depression and anxiety symptoms identified from the participant's baseline and follow-up questionnaire, as well as a note on the tools that the patient received as part of the study. These reports will be sent by mail once the participant completes the follow-up questionnaire.

3 weeks after the summary report is sent, we will mail family physicians a brief survey on their impressions on the intervention and reports sent. See survey in Appendix H.

A discussion group will be held with interested family physicians to explore the potential for implementation of self-care interventions in primary care. Information (see Appendix I) will be shared via social media platforms and through e-bulletins of relevant stakeholder organizations, including through the e-bulletin of *Plakett Services Cliniques*, a consulting firm that co-I Marc Pineault is working with. The bulletin is sent to over 3000 family physicians in Quebec who have previously been in contact with *Plakett Services Cliniques* for operational support for their practice.

Interested family physicians are invited to provide their contact information via an online form on Simple Survey (see link in Appendix I) so that we may reach them for recruitment. The study coordinator will follow-up with the physicians by phone or email to review the online consent form (see Appendix J).

Consenting physicians will be invited to an online discussion group (separate groups will be offered based on language preference and availability) held via Microsoft teams (MSSS platform). Results of the PanDirect and other similar studies will be presented by members of the research team who will co-moderate the discussion. A draft discussion guide is presented in Appendix K.

3.9 Data analysis and statistical power

Data analysis will be conducted and reported according to CONSORT guidelines.[26, 27] All the quantitative analyses will be carry out with SAS University Edition, STATA 15.0 and R Cran software.

The feasibility of the study will be described using the data collected as part of the study log, recruitment, refusal and uptake rates, completion of surveys, and the rates of missing data will be calculated.

Descriptive analyses will compare characteristics of participants associated with participation and completion of follow-up. Incomplete outcome data will constitute an important aspect of our study. Our general approach will be based on intention-to-treat (ITT) and Inverse Probability Weighting (IPW)[48] will be use to handle missing data. Two sensitivity analyzes will be carried out to validate the results obtained by IPW: 1) inclusion of subjects with complete data only; and 2) use of multiple imputation (MI) for missing data.[49, 50] Unless the primary outcome analyses yield different results for completers or MI vs IPW, the secondary analyses will be restricted to completers only.

Baseline imbalance will be assessed by comparing participants randomized to the 2 study arms[51] with respect to 6 pre-specified baseline variables (AGE, SEX, hospital admitted to, COVID-19 DIAGNOSIS, PHQ-9, GAD-7); a maximum of 3 variables with clinically significant differences across arms will be identified.

The comparison of continuous outcomes between intervention and control groups will be based on a simple t-test; linear regression will be used to adjust for baseline imbalance (see above).[52] Similarly, for binary outcome variables (CSQ), Pearson chi-square test and logistic regression[53] will be performed. Effect sizes[54] and 95% confidence intervals (95% CI) will be computed for all the analyses with a continuous outcome, and odds ratio and 95% CI for binary outcomes.

<u>Sample size considerations:</u>

For continuous outcomes (PHQ-9, and GAD-7), a total sample size of n=120 patients will allow us enough power (80%) to detect at least a moderate Cohen's effect size (0.52) between the 2 intervention groups (group equal size, 2-tailed test and alpha 0.05). For the binary outcomes CSQ (75%+) with the same sample size and power, a minimum difference between the two study groups of 20-26% can be detected

4. CONFIDENTIALITY OF PARTICIPANT DATA

All information obtained during the course of the study will be kept confidential. The names of the participants will not appear on the baseline interview or follow-up survey. All files will be kept on a password-protected computer, or in a locked cabinet. Only study identification numbers will be used to identify participants. St. Mary's Hospital Centre Research Ethics Committee may access study records for quality assurance purposes. Materials will be destroyed (paper copies will be shredded and electronic files will be permanently deleted) 5 years after the end of the study.

Research assistants working from home will be bound by St Mary's Research Centre confidentiality agreements. They are required to make phone calls from an environment that mimics their office setting, and allows for calls to be made privately and securely (their phone number will not appear on participants' caller IDs). Any paper forms or notes they may use will have to be locked in a cabinet to which only they have the key. All recruitment/subject logs, digital data entry forms and audio files will be saved on secure, password protected folders on the hospital R drive, and will be accessible from home using the remote Jeton function administered by the CIUSSS.

5. REFERENCES

1. Patten SB, Li Wang J, William JVA, Currie S, Beck CA, Maxwell CJ, et al. Descriptive epidemiology of major depression in Canada. Can J Psychiatry. 2006;51(2):84-90.

2. Zung WWK, Broadhead WE, Roth ME. Prevalence of depressive symptoms in primary care. J Fam Pract. 1993;37(4):337-44.

3. Vonkorff M, Ormel J, Katon W, Lin EHB. Disability and depression among high utilizers of health-care - A longitudinal analysis. Arch Gen Psychiatry. 1992;49(2):91-100.

4. McCusker J, Yaffe M, Lambert SD, Cole M, de Raad M, Belzile E, et al. Unmet needs of family caregivers of hospitalized older adults preparing for discharge home. Chron Illn. 2020;16(2):131-45.

5. McCusker J, Latimer E, Cole M, Ciampi A, Sewitch M. Major depression among medically ill elders contributes to sustained poor mental health in their informal caregivers. Age Ageing. 2007;36(4):400-6.

6. Moussavi S, Chatterji S, Verdes E, Tandon A, Patel V, Ustun B. Depression, chronic diseases, and decrements in health: Results from the world health surveys. Lancet. 2007;370(9590):851-8.

7. Rajkumar RP. COVID-19 and mental health: A review of the existing literature. Asian J Psychiatr. 2020;52:102066.

8. Wang C, Pan R, Wan X, Tan Y, Xu L, Ho CS, et al. Immediate psychological responses and associated factors during the Initial stage of the 2019 Coronavirus disease (COVID-19) epidemic among the general population in China. Int J Environ Res Public Health. 2020;17(5).

9. Kang L, Li Y, Hu S, Chen M, Yang C, Yang BX, et al. The mental health of medical workers in Wuhan, China dealing with the 2019 novel coronavirus. Lancet Psychiatry. 2020;7(3):e14.

10. Wang C, Pan R, Wan X, Tan Y, Xu L, McIntyre RS, et al. A longitudinal study on the mental health of general population during the COVID-19 epidemic in China. Brain Behav Immun. 2020;87:40-8.

11. Findlay LC, Rubab A, Hoken D. Understanding the perceived mental health of Canadians during the COVID-19 pandemic. Health Rep [Internet]. 2020 June 30, 2020. Available from: https://www150.statcan.gc.ca/n1/pub/82-003-x/2020004/article/00003-eng.htm.

12. Lima CKT, Carvalho PMM, Lima I, Nunes J, Saraiva JS, de Souza RI, et al. The emotional impact of Coronavirus 2019-nCoV (new Coronavirus disease). Psychiatry Res. 2020;287:112915.

13. Yang Y, Li W, Zhang Q, Zhang L, Cheung T, Xiang YT. Mental health services for older adults in China during the COVID-19 outbreak. Lancet Psychiatry. 2020;7(4):e19.

14. Hanson J, Percival J, Aldred H, Brownsell S, Hawley M. Attitudes to telecare among older people, professional care workers and informal carers: A preventative strategy or crisis management? Universal Access in the Information Society. 2007;6:193-205.

15. Adler R. The age wave meets the technology wave: broadband and older Americans: SeniorNet 2; 2002 [Available from: http://www.seniornet.org/downloads/broadband.pdf.

16. Bujnowska-Fedak MM, Grata-Borkowska U. Use of telemedicine-based care for the aging and elderly: Promises and pitfalls. Smart Homecare Techn. 2015;3:91-105.

17. Cimperman M, Brencic MM, Trkman P, Stanonik MD. Older adults' perceptions of home telehealth services. Telemed E-Health. 2013;19(10):786-90.

18. National Collaborating Centre for Mental Health. NICE clinical guidelines 91- Depression in adults with a chronic physical health problem: treatment and management. London: National Institute for Health and Clinical Excellence 2009.

19. Fournier L, Roberge P, Brouillet H. Faire face à la dépression au Québec. Protocole de soins à l'intention des intervenants de première ligne. Montréal, QC Centre de recherche du CHUM.; 2012. Available from: https://www.inspq.qc.ca/pdf/publications/1509_FaireFaceDepressionQc_ProtoSoinsInterv1reLigne.pdf.

20. Cuijpers P, Donker T, Van Straten A, Li J, Andersson G. Is guided self-help as effective as face-to-face psychotherapy for depression and anxiety disorders? A systematic review and meta-analysis of comparative outcome studies. Psychol Med. 2010;40(12):1943-57.

21. Gellatly J, Bower P, Hennessy S, Richards D, Gilbody S, Lovell K. What makes self-help interventions effective in the management of depressive symptoms? Meta-analysis and meta-regression. Psychol Med. 2007;37(9):1217-28.

22. McCusker J, Cole M, Yaffe M, Strumpf E, Sewitch M, Sussman T, et al. Adherence to a depression self-care intervention among primary care patients with chronic physical conditions: a randomized controlled trial. Health Educ J. 2016;75(7):767-79.

23. McCusker J, Cole MG, Yaffe M, Strumpf E, Sewitch M, Sussman T, et al. A randomized trial of a depression self-care toolkit with or without lay telephone coaching for primary care patients with chronic physical conditions. Gen Hosp Psychiatry. 2016;37(3):257-65. [Corrigendum: General Hospital Psychiatry, 40: 75-83, 2016].

24. McCusker J, Yaffe M, Faria R, Lambert S, Li M, Poirier-Bisson J, et al. Phase II trial of a depression self-care intervention for adult cancer survivors. Eur J Cancer Care (Engl). 2018;27(1):1-16.

25. McCusker J, Jones J, Li M, Faria R, Yaffe M, Lambert S, et al. Effectiveness of a telephone-supported depression self-management intervention for cancer survivors. [Oral presentation]. 35th CAPO Annual Conference (hosted virtually; July 14-162020.

26. Schulz KF, Altman DG, Moher D. CONSORT 2010 statement: updated guidelines for reporting parallel group randomised trials. J Pharmacol Pharmacother. 2010;1(2):100-7.

27. Zwarenstein M, Treweek S, Gagnier JJ, Altman DG, Tunis S, Haynes B, et al. Improving the reporting of pragmatic trials: an extension of the CONSORT statement. BMJ. 2008;337:a2390.

28. Davidson KW, Goldstein M, Kaplan RM, Kaufmann PG, Knatterud GL, Orleans CT, et al. Evidence-based behavioral medicine: what is it and how do we achieve it? Ann Behav Med. 2003;26:161-71.

29. McCusker J, Beauchamp S, Lambert SD, Proulx R, Yaffe M. Adaptation et implantation d'un outil axé sur le patient pour une amélioration de la planification du congé hospitalier auprès des ainées =

Adapting and implementing a patient centered discharge plan (PCAP) to improve hospital discharge planning for seniors. [Grant]. Ministère de l'Économie, de la Science et de l'Innovation (MÉSI) - Fonds de soutien à l'innovation en santé et en services sociaux (FSISSS): Awarded; 2018. p. $119,500.00CAD.

30. Galvin R, Gilleit Y, Wallace E, Cousins G, Bolmer M, Rainer T, et al. Adverse outcomes in older adults attending emergency departments: a systematic review and meta-analysis of the Identification of Seniors At Risk (ISAR) screening tool. Age Ageing. 2017;46(2):179-86.

31. Katzman R, Brown T, Fuld P, Peck A, Schechter R, Schimmel H. Validation of a short orientation-memory-concentration test of cognitive impairment. Am J Psychiatry. 1983;140(6):734-9.

32. Fillenbaum GG, Heyman A, Wilkinson WE, Haynes CS. Comparison of two screening tests in Alzheimer's disease: the correlation and reliability of the Mini-Mental State Examination and the Modified Blessed Test. Arch Neurol. 1987;44:924-7.

33. McCusker J, Cole M, Lambert S, Yaffe M, Ciampi A, Belzile E. Baseline psychological treatment reduces the effect of coaching in a randomised trial of a depression self-care intervention. Can J Psychiatry. 2017;62(1):67-72.

34. Kroenke K, Spitzer RL, Williams JBW. The PHQ-9: Validity of a brief depression severity measure. J Gen Intern Med. 2001;16:606-13.

35. Manea L, Gilbody S, McMillan D. Optimal cut-off score for diagnosing depression with the Patient Health Questionnaire (PHQ-9): a meta-analysis. Can Med Assoc J. 2012;184(3):E191-6.

36. Lowe B, Kroenke K, Herzog W, Grafe K. Measuring depression outcome with a brief self-report instrument: sensitivity to change of the Patient Health Questionnaire (PHQ-9). J Affect Disord. 2004;81(1):61-6.

37. Thekkumpurath P, Walker J, Butcher I, Hodges L, Kleiboer A, O'Connor M, et al. Screening for major depression in cancer outpatients: the diagnostic accuracy of the 9-item Patient Health Questionnaire. Cancer. 2011;117(1):218-27.

38. van Wilgen CP, Dijkstra PU, Stewart RE, Ranchor AV, Roodenburg JL. Measuring somatic symptoms with the CES-D to assess depression in cancer patients after treatment: comparison among patients with oral/oropharyngeal, gynecological, colorectal, and breast cancer. Psychosomatics. 2006;47(6):465-70.

39. Mitchell AJ, Lord K, Symonds P. Which symptoms are indicative of DSMIV depression in cancer settings? An analysis of the diagnostic significance of somatic and non-somatic symptoms. J Affect Disord. 2012;138(1-2):137-48.

40. Spitzer RL, Kroenke K, Williams JB, Lowe B. A brief measure for assessing generalized anxiety disorder: the GAD-7. Arch Intern Med. 2006;166(10):1092-7.

41. Dhalla S, Kopec JA. The CAGE questionnaire for alcohol misuse: a review of reliability and validity studies. Clin Invest Med. 2007;30(1):33-41.

42. Attkisson CC. Client satisfaction questionnaire (CSQ-8) In: Corcoran K, Fischer J, editors. Measures for clinical practice: a sourcebook. 2. 3rd ed. New York, NY: Free Press; 1987.

43. Sabourin S, Pérusse D, Gendreau P. Les qualités psychométriques de la version canadienne-française du questionnaire de satisfaction du consommateur de services psychothérapeutiques (QSC-8 et QSC-18B) [The Canadian-French version of the client satisfaction questionnaire]. Can J Behav Sci. 1989;21(2):147-59.

44. Bilsker D, Paterson R. Antidepressant skills workbook. Vancouver, British Columbia: Centre for Applied Research in Mental Health and Addiction(CARMHA) and BC Mental Health & Addiction Services (BCMHAS); 2010 [cited 2013 October 31]. Available from: http://www.comh.ca/antidepressant-skills/adult/.

45. Bilsker D, Anderson J, Samra J, Goldner E, Streiner D. Behavioural interventions in primary care: an implementation trial. Can J Commun Ment Health. 2008;27(2):179 -89.

46. Bilsker D, Samra J, Goldner E. Managing Worry. In: Bilsker D, Samra J, Goldner E, editors. Positive Coping with Health Conditions: A Self-Care Workbook. Vancouver: Consortium for Organizational Mental Healthcare; 2009.

47. Lambert SD, Laizner A, Grover S, Belzile E, McCusker J, Moodie E, et al. Internet-based stress management program for patients with cardiovascular disease: piloting a Sequential Multiple Assignment Randomized Trial (SMART). . Poster presented at the International Behavioural Trials Network (IBTN), Montreal (because of COVD-19, conference now online), May 28th – 29th 20202020.

48. Seaman SR, White IR. Review of inverse probability weighting for dealing with missing data. Statistical Methods in Medical Research. 2013;22(3):278-95.

49. Little RJA, Rubin DB. Statistical analysis with missing data. 2nd ed. New York: Wiley; 2001.

50. Rubin DB. Multiple imputation for nonresponse in surveys. New York: John Wiley & Sons; 1987. 1-287 p.

51. Altman DG. Comparability of randomised groups. The Statistician. 1985;34(1):125-36.

52. Neter J, Wasserman W, Kutner MH. Applied linear statistical models Homewood, IL: Irwin; 1985.

53. Hosmer DWJ, Lemeshow S. Applied Logistic Regression. New York: John Wiley & Sons, Inc.; 1989. 307 p.

54. Cohen J. Statistical power analysis for the behavioral sciences. 2nd ed. Hillsdale, NJ: Lawrence Erlbaum Associates, Publishers; 1988. xxi, 567 p. p.

6. APPENDICES

Appendix 0: Information on the 2-1-1 hotline

Appendix A: Recruitment script and screening questions

Appendix B: Consent form

Appendix C: Baseline and follow-up questionnaire

Appendix D: Toolkit contents, description of the algorithm

Appendix E: Coach manual

Appendix F: Fidelity checklist
Appendix G: Summary report for family physicians
Appendix H: Survey for family physician
Appendix I: Invitation information for family physician discussion groups
Appendix J: Consent form for family physician discussion groups
Appendix K: Discussion guide for family physician discussion groups
